# Vaccine-Mediated Immunity Against Dengue and the Potential for Long-Term Protection Against Disease

**DOI:** 10.3389/fimmu.2014.00195

**Published:** 2014-05-06

**Authors:** Mark K. Slifka

**Affiliations:** ^1^Molecular Microbiology and Immunology, Division of Neuroscience, Oregon National Primate Research Center, Oregon Health & Science University, Beaverton, OR, USA

**Keywords:** dengue, antibody, CD8^+^ T cells, CD4^+^ T cells, immunological memory, correlates of immunity, vaccines

## Abstract

It is estimated that over 2.5 billion people are at risk for contracting dengue, a virus responsible for 50–390 million infections in addition to thousands of hospitalizations and deaths each year. There are no licensed vaccines available to combat this pathogen but substantial efforts are underway to develop live-attenuated, inactivated, and subunit vaccines that will protect against each of the four serotypes of dengue. Unfortunately, the results of a recent Phase IIb efficacy trial involving a tetravalent live-attenuated chimeric dengue virus vaccine have raised questions with regard to our current understanding of vaccine-mediated immunity to this important flavivirus. Here, we will briefly summarize these vaccination efforts and discuss the importance of informative *in vivo* models for determining vaccine efficacy and the need to establish a quantitative correlate of immunity in order to predict the duration of vaccine-induced antiviral protection.

## Introduction

Dengue virus (DENV) represents a serious threat to the global community with transmission occurring in over 100 countries (1, 2). Within DENV, there are four distinct serotypes (DENV1, DENV2, DENV3, and DENV4) and each serotype has been found to cause human disease and mortality. DENV infection may result in a spectrum of disease, ranging from acute, debilitating febrile illness [dengue fever (DF)] to severe, life-threatening hemorrhagic disease [dengue hemorrhagic fever; DHF/dengue shock syndrome (DSS)]. Previous estimates indicated that there were 50–100 million cases of DENV infection and 250,000–500,000 cases of DHF/DSS each year, placing over 2.5 billion people at risk (1, 3–5). More recent analysis indicates that the overall burden of DENV could be as high as 390 million infections per year with as many as 96 million demonstrating clinically apparent disease (2). Vector control is currently the only means to reduce the risk of DENV transmission but development of a safe and effective vaccine is urgently needed in order to substantially reduce DENV disease worldwide.

## Failure of CYD-TDV Vaccine Phase 2B Trial

A number of DENV vaccines are in various stages of development with several candidates undergoing testing in early-stage clinical trials (6–12). Of these candidates, the most advanced vaccine is ChimeriVax™, a recombinant flavivirus vaccine technology in which the envelope and PrM proteins of the attenuated vaccine strain of yellow fever 17D (YFV-17D) have been replaced with the proteins of each serotype of DENV. A tetravalent formulation containing four chimeric yellow fever vaccine strains of DENV, termed, CYD-TDV, has been developed and the results of the Phase IIb efficacy trial involving 4002 subjects have been reported (13). Since YFV-17D is a highly immunogenic and successful vaccine, it was generally anticipated that the recombinant CYD-TDV vaccine, would induce bona fide flavivirus-specific immune responses that would lead to strong and durable protective immunity against DENV infection. Unfortunately, overall vaccine efficacy was only 30% and differed by serotype. The majority of DENV infections were of the DENV2 serotype (48 cases in the per-protocol analysis), and it showed the lowest efficacy (9%). The highest vaccine efficacy was observed with DENV4 (100% efficacy based on 4 cases), followed by DENV3 (75% efficacy based on 3 cases), and DENV1 (56% efficacy based on 19 cases). The numbers of DENV cases in this study were relatively small and more data are needed to verify these potential rates of serotype-specific vaccine-mediated protection. Ongoing Phase III trials involving 30,000 volunteers have been initiated (NCT01373281 and NCT01374516) and these should provide more definitive data on vaccine efficacy across the four DENV serotypes.

The lack of observed efficacy in the CYD-TDV vaccine trial was unexpected since seroprevalence rates were high at baseline (e.g., ~70% seropositive to at least one of the four different DENV serotypes) and seroconversion to DENV2 reached 87% in the vaccinated group within 28 days after the first vaccination and 99% after the second vaccination (13). The conundrum of high vaccine immunogenicity but low protective efficacy has led to many questions regarding the lack of protection and the potential factors that may be involved. Vaccine interference from pre-existing anti-flavivirus immunity within the DENV-endemic population is one possibility or maybe the neutralizing assays used to measure immunogenicity are not able to distinguish between protective and non-protective levels of immunity. Alternatively, T cell-mediated immunity may be important for DENV-specific protection and antiviral T cell responses were not measured in this study. Overcoming pre-existing immunity is known to be a problem for the live-attenuated DENV vaccines and even booster vaccinations must be separated by long intervals (e.g., 0, 6, 12-month vaccination schedule) (14, 15) or the “boosting” effect of secondary or tertiary vaccination is dampened by the immunity generated by the prior vaccinations. Vaccine interference is a common problem among all of the tetravalent live-attenuated DENV vaccine formulations, resulting in biased neutralizing antibody responses to some, but not all DENV serotypes until multiple vaccinations have been performed (16). Within an endemic community, it is possible that one or more of the vaccine strains of DENV are inhibited from efficient replication in the host and result in reduced induction of homotypic neutralizing antibodies to a broader number of DENV serotypes. Moreover, the cohorts at the site of the CYD-TDV trial also had high levels of pre-existing immunity to other flaviviruses (e.g., 78–80% seropositive for JEV) (13) and it is unclear if immunity to multiple flaviviruses is having a positive or negative impact on vaccine efficacy in the field. In addition to live, attenuated DENV vaccines such as CYD-TDV, there are several non-replicating vaccine approaches currently in clinical development including formalin-inactivated whole virion vaccines (NCT01502735, NCT01666652, and NCT01702857) and DENV envelope subunit protein vaccines (NCT00936429 and NCT01477580) and it will be interesting to learn if these non-replicating vaccine approaches suffer the same challenges as the live, attenuated DENV vaccines or if they are able to overcome viral interference in DENV-endemic communities.

Dengue virus-specific neutralizing assays are a key measurement of vaccine-induced antiviral immunity but there is considerable debate over the best approach to performing these assays. The 50% plaque-reduction neutralizing test (PRNT_50_) assay used to measure antiviral immunity in the CYD-TDV Phase IIb trial has come into question since it was performed in Vero cells (16) and some have proposed that DENV strains should be grown in other cell types or that primary DENV strains isolated directly from acutely infected DENV patients be used in the analysis (17). It may not be feasible to perform standardized neutralization assays under GLP compliance using direct DENV isolates from human serum but these questions nevertheless further illustrate the point that despite the publication of a WHO guidance document (18), there is still little consensus among the scientific community on how neutralizing titers to DENV should be performed or which DENV strains should be used in this crucial analysis of antiviral immunity. Although the Phase IIb trial followed published WHO guidelines on performing DENV neutralization assays (18), these immunological assays were not performed with reference strains of each DENV serotype, but were instead performed with the individual chimeric CYD-TDV vaccine strains of virus that represented each DENV serotype (13). This could be problematic in the interpretation of seroconversion as well as in determining the true magnitude of DENV-specific antibody responses. For example, vaccine-induced neutralizing antibodies to the homologous CYD-TDV vaccine virus strains are typically 2-fold to >10-fold higher then the results obtained when non-recombinant wild-type strains of DENV of the same serotype are used for determining neutralizing titers (19). These results are not unique to CYD-TDV since similar results have been observed with a chimeric YFV vector expressing JEV envelope proteins (20) and a chimeric DENV vector expressing WNV envelope proteins (21). In each case, immunization with the vaccine strain of recombinant virus elicited higher antibody responses to the vaccine strain of virus than to the wild-type target strain of flavivirus (22). This means that in terms of the CYD-TDV Phase IIb trial, the high serotype-specific seroconversion rates observed against CYD-TDV strains of recombinant virus may have been lower if reference strains of wild-type DENV had been used in the PRNT_50_ assays.

## Role of Vaccine-Induced T Cells in Protection Against Flavivirus Infection

With DENV (23) or West Nile virus (24–26), CD8^+^ T cells can protect mice against viral infection, but pre-existing T cell memory is not absolutely required for protection since passive transfer of immune serum or neutralizing monoclonal antibodies can also mediate protection against lethal challenge (27, 28). In the AG129 mouse model of DENV infection, antiviral antibodies appear to play a greater role than T cells in protection against DENV challenge (29) and administration of neutralizing monoclonal antibodies can provide full protective immunity (27). However, the role of human T cell-mediated immunity in protection or pathogenesis during flavivirus infection remains unclear. For instance, although higher antiviral T cell responses to DENV have been found during DHF (30), it is uncertain if the antiviral T cell response is involved with causing disease or if it is instead an epiphenomenon in response to the higher viral load associated with DHF. Higher antiviral T cell responses/IFNγ production have also been associated with lower disease during DENV infection (31, 32), although it is unknown if this is due to a direct relationship to antiviral T cell immunity or if it is possible that higher antiviral T cell responses represent a biomarker indicative of better antiviral antibody responses. Primary infection with YFV-17D (33–36) or DENV (30, 37) induces strong antiviral T cell responses that are mounted against all viral proteins, although most are directed toward non-structural proteins. Most clinical studies are limited to measuring associations and it is often difficult to determine a direct cause-and-effect relationship between T cell responses and viral burden because normal vaccine-mediated antiviral immune responses include induction of both humoral and cellular immunity. One way to directly determine if vaccine-induced human T cell responses play a role in antiviral immunity to flaviviruses is to immunize human subjects with a vaccine that elicits antiviral CD4^+^ and CD8^+^ T cell responses in the absence of a virus-specific neutralizing antibody response. Interestingly, these studies have been performed during the early clinical testing of the ChimeriVax™ vaccine platform (38, 39) (Table 1). ChimeriVax is constructed with eight YFV-17D non-structural proteins but the YFV-17D envelope and PrM proteins are replaced with the surface proteins from another flavivirus (8). In two studies, the envelope proteins and the associated neutralizing epitopes of YFV-17D were replaced with the envelope proteins (and their associated virus-specific neutralizing epitopes) of DENV2 (39) or JEV (38) and the role of pre-existing YFV-17D-specific T cells in antiviral immunity was determined (Table 1). When YFV-17D-naïve subjects were vaccinated/infected with YFV-17D (10^5^ PFU/dose), 100% of the vaccinees became viremic. Likewise, when YFV-17D naïve individuals were infected with ChimeriVax vaccine strains expressing the DENV2 envelope/PrM proteins (YFV-DENV2; 10^5^ PFU/dose) or JEV envelope/PrM proteins (YFV-JEV; 10^5^ or 10^4^ PFU/dose), viremia was observed in 57.1, 83.3, and 100% of subjects, respectively. This demonstrates that these chimeric viruses maintained viral fitness in their human host and readily induced viremia in flavivirus-naïve individuals with a measurable peak and duration of systemic infection. Vaccination of YFV-17D-immune subjects proved to be an insightful experiment because these individuals presumably have pre-existing antiviral CD4^+^ and CD8^+^ T cells to the yellow fever non-structural proteins encoded in the ChimeriVax vector, but would lack neutralizing antibody responses because the YFV-17D envelope and PrM genes in these recombinant viruses have been replaced by DENV2 or JEV envelope/PrM genes. Although YFV-17D (10^5^ PFU/dose) failed to induce detectable viremia in YFV-17D-immune subjects (0% viremic), YFV-DENV2 (10^5^ PFU/dose), and YFV-JEV (10^5^ PFU/dose) caused viremia with infection rates that were similar to that observed in YFV-17D-naïve subjects. Even the lower dose of YFV-JEV (10^4^ PFU/dose) infected 100% of the YFV-17D-immune subjects, indicating that antiviral T cell memory was insufficient for inhibiting low-dose viral challenge (Table 1). Together, these data suggest that pre-existing CD8^+^ and CD4^+^ memory T cell responses to 8/10 of the flavivirus proteins, including all of the non-structural proteins, failed to protect against flavivirus infection in an experimental model in which antiviral neutralizing antibody responses were absent.

**Table 1:** Analysis of viremia in human subjects with or without pre-existing antiviral T cell memory*.

YFV-17D-Naïve					YFV-17D-Immune			
Challenge virus	YFV-17D 10^5^ PFU	YFV-DENV2 10^5^ PFU	YFV-JEV 10^5^ PFU	YFV-JEV 10^4^ PFU	YFV-17D 10^5^ PFU	YFV-DENV2 10^5^ PFU	YFV-JEV 10^5^ PFU	YFV-JEV 10^4^ PFU
*n*	5	14	6	6	6	14	6	6
Viremia (%)	100	57.1	83.3	83.3	0	78.6	83.3	100
AUC	56	20.7	21.7	48.3	0	50.4	58.3	50

*Two clinical studies were conducted to determine if pre-existing YFV-specific immunity would impact virus replication upon challenge with YFV-17D or chimeric versions of YFV-17D in which the envelope and PrM proteins of YFV-17D were replaced by the envelope and PrM proteins of either DENV2 (39) or JEV (38). The chimeric flaviviruses, YFV-DENV2 and YFV-JEV, have the same eight non-structural proteins as YFV-17D and contain the same CD4^+^ and CD8^+^ T cell epitopes, but can no longer be neutralized by YFV-17D-specific antibodies, thus providing the opportunity to measure T cell-mediated protection in the absence of neutralizing antibodies to the viral structural proteins; *n*, number of subjects per group; PFU, plaque forming units; AUC, area under the curve.

Virus-specific T cells cannot block infection *per se* (this is best accomplished by neutralizing antibodies), but may be involved with reducing viral load once an infection has occurred. However, pre-existing T cell memory did not reduce the peak level of viremia or lower the duration of viremia by chimeric YFV-JEV or YFV-DENV2 (38, 39). This information was captured in the area under the curve (AUC) measurements that combined the magnitude and duration of viremia measurements and based on this assessment, pre-existing T cell memory in YFV-17D-immune subjects did not play a measurable role in reducing viral load after chimeric YFV-17D-based flavivirus infection (Table 1). In contrast, YFV-17D-immune subjects were fully protected against YFV-17D that express the homologous envelope proteins (38, 40). The protection in this case may be largely due to neutralizing antibodies since prior studies have demonstrated that adoptive transfer of immune serum alone provides partial to full protective immunity against lethal YFV in rhesus macaques (RM) (41), hamsters (42), and immunodeficient mice (43).

Although vaccine-induced T cell memory failed to prevent viremia, one would anticipate that another contribution of cellular immunity would be to modify disease upon flavivirus reinfection. However, amelioration of disease symptoms was not observed; following infection with YFV-DENV2, the incidence of myalgia, arthralgia, rash, and rigors was higher in YFV-17D-immune subjects compared to YFV-17D-naïve subjects (39) and following YFV-JEV infection, the only subject with a high fever (102.1°F) belonged to the YFV-17D-immune group. Of the other two cases of low-grade fever considered by the investigators to be possibly related to vaccination, these also occurred in the YFV-17D-immune group (38). It is important to keep in mind that these are relatively small clinical studies and it is possible that antiviral T cell memory plays a more substantial role in protection against wild-type flaviviruses or that they may function in a manner that was not measured in these clinical assessments. However, based on this work there appears to be little evidence that pre-existing vaccine-induced T cell memory is involved with prevention of secondary flavivirus infection, dissemination, or early disease progression. In addition, because YFV-17D-immune subjects would be expected to have pre-existing antibodies to as many as eight non-structural YFV proteins that are found in the recombinant YFV-JEV and YFV-DENV2 viruses, this work also suggests that non-neutralizing antibodies to these viral proteins are unlikely to play a major role in vaccine-mediated protection against flavivirus infection.

## Concerns of Vaccine-Induced Antibody Dependent Enhancement

The pathogenesis of DENV is complex and there has been considerable concern that vaccine-induced antibody dependent enhancement (ADE) of DENV infection could result in exacerbated disease among vaccinated individuals who have only partial immunity or low-level heterotypic immunity to secondary DENV infection (44–46). ADE is a phenomenon in which non-neutralizing antibodies or sub-neutralizing levels of virus-specific antibodies result in enhanced infection of Fc receptor-bearing cells (e.g., macrophages, monocytes) (44, 45, 47). Fortunately, long-term monitoring of vaccinees in DENV-endemic countries has not revealed evidence of ADE. For example, one group found that 4/113 (3.5%) vaccine recipients had been hospitalized with DENV within 6.8 years after DENV vaccination whereas 14/226 (6.2%) unvaccinated, age-matched, and location-matched children were hospitalized due to DENV (45). Perhaps the most compelling evidence for a lack of vaccine-mediated ADE comes from the CYD-TDV Phase IIb trial (13). This study provided an example of measurable immunogenicity but low protective efficacy and would be expected to result in the highest likelihood of ADE. However, despite incomplete vaccine-mediated protection against the four serotypes of DENV (and a non-protective immune response to the circulating strain of DENV2), analysis of 2600 vaccinated children monitored for 2 years after vaccination (i.e., 5200 person-years) showed no increase in the rate or clinical severity of DENV infection among the vaccinated population. This is important safety information and provides further support for continued development of an effective DENV vaccine.

## Neutralizing Antibodies and the Duration of Vaccine-Mediated Immunity

**Figure 1:**
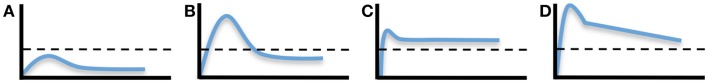
**Dynamics and duration of vaccine-induced immunity**. The development of pathogen-specific immunity after vaccination can follow a number of different kinetic models. **(A)** Development of a measurable immune response can be determined, but if it does not reach above the protective threshold (indicated by the dashed line), then measurable immunity will note equate to protective immunity. Short-lived protective immunity **(B)** is common following primary immunization and is one of the reasons why most vaccines require booster vaccination. Long-lived protective immunity may be achieved by durable or nearly steady-state levels of immunity **(C)** or through the development of strong but rapidly declining immunity **(D)**, if the starting point begins high in reference to the protective threshold. It is important to note that the protective threshold will differ by pathogen or disease and an immunological correlate of protection must be known in order to extrapolate the potential durability of a particular vaccine-mediated immune response.

It is often difficult to estimate how long protective vaccine-mediated immunity will last unless (a) the correlate of immunity has been established and (b) the levels of immunity are measured in longitudinal or cross-sectional studies for a prolonged period of time. For example, if neutralizing antibodies represent the correlate of immunity and the protective threshold is determined to be a PRNT_50_ of 10, then measuring the magnitude and duration of PRNT_50_ titers over time will provide valuable information on the durability of protective immunity (Figure 1). In some cases, vaccination will elicit low levels of immunity that are measurable, but reside below the threshold needed for protection (Figure 1A). An example of this would be the small subset of individuals who receive the MMR vaccine and develop anti-measles antibodies that are readily detected by ELISA, but are below the protective threshold of 0.2 IU/mL (48, 49). Alternatively, vaccines may elicit short-term protective immunity in which antibodies remain above the threshold of protection for a brief period of time before declining below the protective threshold (Figure 1B). There are several examples of vaccines that fall into this category including the acellular pertussis vaccine that provides 98% protection during the first year after completing the primary vaccination series, but then declines steadily to 71% protection by 5 years post-vaccination (50). Live, attenuated vaccines such as the pediatric varicella zoster vaccine also require two doses of virus because, although protective immunity is high shortly after vaccination, the levels of protection wane gradually after the first dose, resulting in significantly higher break-through cases of varicella within 5 years after primary vaccination (51). Longitudinal analysis of DENV1-specific neutralizing titers during CYD-TDV vaccination provides another example of this type of short-lived immunity. Although seroconversion to DENV1 was low after primary vaccination (12.1% seroconversion), after secondary vaccination about 70% of subjects had seroconverted. However, within 4 months after the second vaccination, ~40% of subjects remained seropositive and the residual geometric mean titer of DENV1-specific antibodies appeared to have declined to below a PRNT_50_ = 10 (52). Rapidly waning immunity after vaccination with a live, attenuated DENV vaccine is not unique to CYD-TDV since vaccination with a tetravalent PDK-attenuated DENV vaccine also elicited detectable antibody titers after two vaccinations that decayed rapidly and in some instances declined to below the cut-off value of 1:10 by the PRNT assay (45). Together, this illustrates the point that booster vaccination with live-attenuated CYD-TDV (and other DENV vaccines) is not only required to increase the breadth of serotype-specific neutralizing antibody responses but, similar to vaccines against other pathogens (e.g., MMR, acellular pertussis, varicella), booster vaccinations are important for inducing immune responses that can be maintained above a protective threshold for a prolonged period of time (53).

In contrast to these examples of non-protective or short-lived immunity, long-lived vaccine-mediated immunity can be achieved by at least two mechanisms; induction of an immune response that is long-lived and maintained at a plateau above the protective threshold (Figure 1C) or induction of immunity that may decline at a rapid rate but still be maintained above the protective threshold for a prolonged period of time if it begins at a high initial starting point (Figure 1D). Natural infection with measles or mumps is known to induce long-lived immunity that is often maintained above the protective threshold for many years or possibly for life (49). Vaccination with the live, attenuated yellow fever vaccine is also thought to induce life-long immunity (53). However, closer examination of published studies examining the durability of immunity following yellow fever vaccination indicates that it may reflect a combination of Figures 1B and 1C since about 60–70% of vaccinated subjects maintain durable virus-specific neutralizing antibodies above the protective threshold (i.e., Figure 1C) whereas ~30–40% of vaccinees have neutralizing antibody titers that decline to below the protective threshold within 5–10 years after vaccination (i.e., Figure 1B) (53–55). Compared to most types of viral infection, the duration of immunity against tetanus is relatively short-lived with ~11-year half-life (49, 56). However, since the current five-dose tetanus vaccination regimen induces relatively high titers of tetanus-specific neutralizing antibody, protection is likely maintained above the protective threshold of 0.01 IU/mL for decades, despite having a more rapid decay rate over time (Slifka, manuscript submitted). There are several DENV vaccines under development (live/attenuated, inactivated whole virus, subunit envelope protein, and plasmid DNA) and it remains to be seen if these different approaches to DENV vaccination elicit different levels and duration of immunity. However, to get to the heart of the question pertaining to the duration of vaccine-mediated protection, the immunological correlate of protection to each DENV serotype will need to be identified.

## Defining a Correlate of Protective Immunity

A major caveat to the development of a vaccine against DENV is that the immunological correlate of protection against DENV infection is not currently known. It is possible that a titer of 1:10 may be readily detectable, but may still reside below the protective threshold needed to prevent infection or reduce DENV-associated disease in the clinical setting. Defining a correlate of protective immunity is important especially when examining the durability of vaccine-induced protection (Figure 1). If an appropriate animal model exists, then the correlate of immunity can be determined experimentally. For example, RM are highly susceptible to yellow fever and the correlate of immunity to this virus was first identified by vaccinating RM with graded doses of YFV-17D, followed 20 weeks later by infection with a lethal dose of virulent YFV-Asibi. Approximately 94% of vaccinated animals with a pre-existing log neutralizing index (LNI) of ≥0.7 were protected from lethal infection whereas 91% of animals with <0.7 LNI succumbed to yellow fever infection (57). In this case, RM develop disease that is similar to severe human yellow fever and this is likely one of the reasons why the correlate of immunity to yellow fever (LNI ≥ 0.7) has gained wide acceptance and has been used successfully in the field. Neutralizing antibodies are also believed to be the major component of vaccine-mediated immunity against DENV and protection against DENV viremia in non-human primates (NHP) is associated with a PRNT_50_ titer of ≥10 (15). However, most of the DENV-vaccinated animals in this study (23/24; 96%) had neutralizing antibody titers that were ≥20 at the time of DENV challenge (note; 83% had PRNT_50_ ≥40) and so the cut-off value of PRNT_50_ ≥10 for defining protective immunity may still be an open question. Bearing in mind that DENV strains do not replicate to high titers or cause clinical disease in NHP, it is also difficult to extrapolate to the levels of immunity that might be required to protect against more severe human disease. As shown in the CYD-TDV Phase IIb vaccine trial, detection of a measurable level of antiviral antibodies (i.e., PRNT_50_ = 10) does not necessarily equate to a protective level of neutralizing antibodies (13).

To better understand the mechanisms of protective immunity against DENV in the absence of a robust animal model, a human challenge model of DF is being developed (32, 58–61). Well-characterized strains of DENV1 and DENV3 that were originally tested as vaccine candidates were found to elicit fever and mild dengue disease in early clinical trials and have now provided important information on DENV host–pathogen interactions (32, 58, 60) with the opportunity to directly test vaccine efficacy and determine immunological correlates of immunity. Following infection with DENV1-Ch (DENV1 45AZ5 PDK-0), both of the unvaccinated control subjects developed fever and dengue-associated illness lasting 2–6 days. In contrast, none of the five subjects who had previously received a live, attenuated tetravalent DENV vaccine showed disease symptoms. At the time of challenge, their DENV1-specific neutralizing PRNT_50_ titers were 415, 235, 451, 198, and <10. This indicates that DENV vaccination can protect against DENV1-Ch, but in this small group of subjects there were no break-through cases lacking protection and it was therefore not possible to estimate a correlate of vaccine-mediated immunity against DENV1 disease. In another series of experiments involving experimental infection with DENV3-Ch (DENV3 CH53489 cl 24/28 PDK-0), both unvaccinated control subjects developed fever and dengue-associated illness and 3/5 TDV-vaccinated subjects also developed DF. The three vaccinated subjects who developed mild DF had pre-existing PRNT_50_ titers of <10, 19, and 16, whereas the two vaccinated subjects who were protected from disease symptoms had pre-existing titers of 57 and 116. Based on these early results, this work would suggest that a DENV3-specific neutralizing titer of >20 or >50 may be necessary to protect against clinical DF. Further studies are needed to develop a model for DENV2 and DENV4 (60) and more vaccinated subjects may need to be enrolled with a range of pre-existing antibody titers in order to verify and refine the cut-off value for protection from DENV disease in this challenge model. Alternatively, another useful approach would be to coordinate large seroepidemiology studies of vaccinated subjects in DENV-endemic areas. If serum antibody titers were measured annually and cross-referenced to cases of clinical DENV illness/hospitalization, then it may be possible to extrapolate a vaccine-induced correlate of immunity that distinguishes between protective and non-protective immune responses. Once an immunological correlate can be determined for each of the DENV serotypes, then analysis of the neutralizing titers of DENV vaccines could be measured against this benchmark in order to better predict potential protective efficacy in Phase III trials.

## Conclusion

Although an effective DENV vaccine has not yet reached the market, there are several candidates currently in clinical trial and it is likely that at least one or more of these vaccine platforms will provide protective immunity against this important, yet previously neglected disease. Although the current frontrunner, CYD-TDV, has yet to demonstrate effective protection in Phase IIb field trials, there is still hope that the Phase III trials will be successful. Because the CYD-TDV vaccine elicited only weak/partial immunity, there was concern that ADE would be a factor and one successful outcome of this study is that there was no evidence for exacerbated DENV disease among vaccinated children, which in itself is an important step forward in terms of identifying risk factors during DENV vaccine development. Despite induction of antiviral T cell responses to both structural and non-structural proteins, flavivirus-specific T cell memory in humans appears to play a relatively subordinate role in protection against reinfection (7, 38, 62). Antiviral T cells may play a more important role during primary viral infection than during vaccine-mediated immune responses to secondary infection (7, 62) and it is likely that a specific level of serotype-specific neutralizing antibodies will be found as an immunological correlate of immunity following vaccination. Once an immunological correlate of vaccine-mediated protection is identified, then this will provide the opportunity for more quantitative assessment of current and future “next-generation” DENV vaccines in addition to providing a benchmark for determining the duration of protective immunological memory after vaccination.

## Conflict of Interest Statement

OHSU and Dr. Mark K. Slifka, have a financial interest in Najít Technologies, Inc., a company that is developing a new tetravalent dengue virus vaccine using a hydrogen peroxide-based inactivation approach. This potential individual and institutional conflict of interest has been reviewed and managed by OHSU.

## References

[1] World Health Organisation. Dengue and Dengue Haemorrhagic Fever. Fact sheet No. 117. Geneva: WHO (2014).

[2] Bhatt S Gething PW Brady OJ Messina JP Farlow AW Moyes CL The global distribution and burden of dengue. Nature (2013) 496(7446):504–7 10.1038/nature12060 23563266 PMC3651993

[3] Halstead SB Pathogenesis of dengue: challenges to molecular biology. Science (1988) 239:476–81 10.1126/science.3277268 3277268

[4] Monath TP Dengue: the risk to developed and developing countries. Proc Natl Acad Sci U S A (1994) 91(7):2395–400 10.1073/pnas.91.7.2395 8146129 PMC43378

[5] Thomas SJ Endy TP Vaccines for the prevention of dengue: development update. Hum Vaccin (2011) 7(6):674–84 21508679 10.4161/hv.7.6.14985

[6] Lam SK Challenges in reducing dengue burden; diagnostics, control measures and vaccines. Expert Rev Vaccines (2013) 12(9):995–1010 10.1586/14760584.2013.824712 24053394

[7] Murphy BR Whitehead SS Immune response to dengue virus and prospects for a vaccine. Annu Rev Immunol (2011) 29:587–619 10.1146/annurev-immunol-031210-101315 21219187

[8] Guy B Barrere B Malinowski C Saville M Teyssou R Lang J From research to phase III: preclinical, industrial and clinical development of the Sanofi Pasteur tetravalent dengue vaccine. Vaccine (2011) 29(42):7229–41 10.1016/j.vaccine.2011.06.094 21745521

[9] Coller BA Clements DE Dengue vaccines: progress and challenges. Curr Opin Immunol (2011) 23(3):391–8 10.1016/j.coi.2011.03.005 21514129

[10] Osorio JE Huang CY Kinney RM Stinchcomb DT Development of DENVax: a chimeric dengue-2 PDK-53-based tetravalent vaccine for protection against dengue fever. Vaccine (2011) 29(42):7251–60 10.1016/j.vaccine.2011.07.020 21777638 PMC4592106

[11] Danko JR Beckett CG Porter KR Development of dengue DNA vaccines. Vaccine (2011) 29(42):7261–6 10.1016/j.vaccine.2011.07.019 21777640

[12] Lindow JC Durbin AP Whitehead SS Pierce KK Carmolli MP Kirkpatrick BD Vaccination of volunteers with low-dose, live-attenuated, dengue viruses leads to serotype-specific immunologic and virologic profiles. Vaccine (2013) 31(33):3347–52 10.1016/j.vaccine.2013.05.075 23735680 PMC3777849

[13] Sabchareon A Wallace D Sirivichayakul C Limkittikul K Chanthavanich P Suvannadabba S Protective efficacy of the recombinant, live-attenuated, CYD tetravalent dengue vaccine in Thai schoolchildren: a randomised, controlled phase 2b trial. Lancet (2012) 380(9853):1559–67 10.1016/S0140-6736(12)61428-7 22975340

[14] Guy B Barban V Mantel N Aguirre M Gulia S Pontvianne J Evaluation of interferences between dengue vaccine serotypes in a monkey model. Am J Trop Med Hyg (2009) 80(2):302–11 19190230

[15] Guirakhoo F Pugachev K Zhang Z Myers G Levenbook I Draper K Safety and efficacy of chimeric yellow fever-dengue virus tetravalent vaccine formulations in nonhuman primates. J Virol (2004) 78(9):4761–75 10.1128/JVI.78.9.4761-4775.2004 15078958 PMC387722

[16] Halstead SB Identifying protective dengue vaccines: guide to mastering an empirical process. Vaccine (2013) 31(41):4501–7 10.1016/j.vaccine.2013.06.079 23896423

[17] Chokephaibulkit K Perng GC Challenges for the formulation of a universal vaccine against dengue. Exp Biol Med (Maywood) (2013) 238(5):566–78 10.1177/1535370212473703 23856907

[18] Roehrig JT Hombach J Barrett AD Guidelines for plaque-reduction neutralization testing of human antibodies to dengue viruses. Viral Immunol (2008) 21(2):123–32 10.1089/vim.2008.0007 18476771

[19] Guirakhoo F Pugachev K Arroyo J Miller C Zhang ZX Weltzin R Viremia and immunogenicity in nonhuman primates of a tetravalent yellow fever-dengue chimeric vaccine: genetic reconstructions, dose adjustment, and antibody responses against wild-type dengue virus isolates. Virology (2002) 298(1):146–59 10.1006/viro.2002.1462 12093182

[20] Monath TP Levenbook I Soike K Zhang ZX Ratterree M Draper K Chimeric yellow fever virus 17D-Japanese encephalitis virus vaccine: dose-response effectiveness and extended safety testing in rhesus monkeys. J Virol (2000) 74(4):1742–51 10.1128/JVI.74.4.1742-1751.2000 10644345 PMC111650

[21] Durbin AP Wright PF Cox A Kagucia W Elwood D Henderson S The live attenuated chimeric vaccine rWN/DEN4Delta30 is well-tolerated and immunogenic in healthy flavivirus-naive adult volunteers. Vaccine (2013) 31(48):5772–7 10.1016/j.vaccine.2013.07.064 23968769 PMC3833717

[22] Amanna IJ Slifka MK Current trends in West Nile virus vaccine development. Expert Rev Vaccines (2014) 13(5):589–608 10.1586/14760584.2014.906309 24689659 PMC4279923

[23] Yauch LE Zellweger RM Kotturi MF Qutubuddin A Sidney J Peters B A protective role for dengue virus-specific CD8+ T cells. J Immunol (2009) 182(8):4865–73 10.4049/jimmunol.0801974 19342665 PMC2674070

[24] Shrestha B Diamond MS Role of CD8+ T cells in control of West Nile virus infection. J Virol (2004) 78(15):8312–21 10.1128/JVI.78.15.8312-8321.2004 15254203 PMC446114

[25] Wang Y Lobigs M Lee E Mullbacher A CD8+ T cells mediate recovery and immunopathology in West Nile virus encephalitis. J Virol (2003) 77(24):13323–34 10.1128/JVI.77.24.13323-13334.2003 14645588 PMC296062

[26] Brien JD Uhrlaub JL Nikolich-Zugich J Protective capacity and epitope specificity of CD8(+) T cells responding to lethal West Nile virus infection. Eur J Immunol (2007) 37(7):1855–63 10.1002/eji.200737196 17559175

[27] Shrestha B Brien JD Sukupolvi-Petty S Austin SK Edeling MA Kim T The development of therapeutic antibodies that neutralize homologous and heterologous genotypes of dengue virus type 1. PLoS Pathog (2010) 6(4):e1000823 10.1371/journal.ppat.1000823 20369024 PMC2848552

[28] Oliphant T Engle M Nybakken GE Doane C Johnson S Huang L Development of a humanized monoclonal antibody with therapeutic potential against West Nile virus. Nat Med (2005) 11(5):522–30 10.1038/nm1240 15852016 PMC1458527

[29] Kyle JL Balsitis SJ Zhang L Beatty PR Harris E Antibodies play a greater role than immune cells in heterologous protection against secondary dengue virus infection in a mouse model. Virology (2008) 380(2):296–303 10.1016/j.virol.2008.08.008 18774583 PMC2590773

[30] Duangchinda T Dejnirattisai W Vasanawathana S Limpitikul W Tangthawornchaikul N Malasit P Immunodominant T-cell responses to dengue virus NS3 are associated with DHF. Proc Natl Acad Sci U S A (2010) 107(39):16922–7 10.1073/pnas.1010867107 20837518 PMC2947904

[31] Hatch S Endy TP Thomas S Mathew A Potts J Pazoles P Intracellular cytokine production by dengue virus-specific T cells correlates with subclinical secondary infection. J Infect Dis (2011) 203(9):1282–91 10.1093/infdis/jir012 21335561 PMC3069729

[32] Gunther VJ Putnak R Eckels KH Mammen MP Scherer JM Lyons A A human challenge model for dengue infection reveals a possible protective role for sustained interferon gamma levels during the acute phase of illness. Vaccine (2011) 29(22):3895–904 10.1016/j.vaccine.2011.03.038 21443963

[33] Miller JD van der Most RG Akondy RS Glidewell JT Albott S Masopust D Human effector and memory CD8+ T cell responses to smallpox and yellow fever vaccines. Immunity (2008) 28(5):710–22 10.1016/j.immuni.2008.02.020 18468462

[34] Akondy RS Monson ND Miller JD Edupuganti S Teuwen D Wu H The yellow fever virus vaccine induces a broad and polyfunctional human memory CD8+ T cell response. J Immunol (2009) 183(12):7919–30 10.4049/jimmunol.0803903 19933869 PMC3374958

[35] James EA LaFond RE Gates TJ Mai DT Malhotra U Kwok WW Yellow fever vaccination elicits broad functional CD4+ T cell responses that recognize structural and nonstructural proteins. J Virol (2013) 87(23):12794–804 10.1128/JVI.01160-13 24049183 PMC3838168

[36] Blom K Braun M Ivarsson MA Gonzalez VD Falconer K Moll M Temporal dynamics of the primary human T cell response to yellow fever virus 17D as it matures from an effector- to a memory-type response. J Immunol (2013) 190(5):2150–8 10.4049/jimmunol.1202234 23338234

[37] Weiskopf D Angelo MA de Azeredo EL Sidney J Greenbaum JA Fernando AN Comprehensive analysis of dengue virus-specific responses supports an HLA-linked protective role for CD8+ T cells. Proc Natl Acad Sci U S A (2013) 110(22):E2046–53 10.1073/pnas.1305227110 23580623 PMC3670335

[38] Monath TP McCarthy K Bedford P Johnson CT Nichols R Yoksan S Clinical proof of principle for ChimeriVax: recombinant live, attenuated vaccines against flavivirus infections. Vaccine (2002) 20(7–8):1004–18 10.1016/S0264-410X(01)00457-1 11803060

[39] Guirakhoo F Kitchener S Morrison D Forrat R McCarthy K Nichols R Live attenuated chimeric yellow fever dengue type 2 (ChimeriVax-DEN2) vaccine: phase I clinical trial for safety and immunogenicity: effect of yellow fever pre-immunity in induction of cross neutralizing antibody responses to all 4 dengue serotypes. Hum Vaccin (2006) 2(2):60–7 10.4161/hv.2.2.2555 17012873

[40] Reinhardt B Jaspert R Niedrig M Kostner C L’Age-Stehr J Development of viremia and humoral and cellular parameters of immune activation after vaccination with yellow fever virus strain 17D: a model of human flavivirus infection. J Med Virol (1998) 56(2):159–67 10.1002/(SICI)1096-9071(199810)56:23.0.CO;2-B 9746073

[41] Sawyer WA Persistence of yellow fever immunity. J Prev Med (1931) 5:413–28

[42] Julander JG Trent DW Monath TP Immune correlates of protection against yellow fever determined by passive immunization and challenge in the hamster model. Vaccine (2011) 29(35):6008–16 10.1016/j.vaccine.2011.06.034 21718741 PMC3148314

[43] Thibodeaux BA Garbino NC Liss NM Piper J Schlesinger JJ Blair CD A humanized IgG but not IgM antibody is effective in prophylaxis and therapy of yellow fever infection in an AG129/17D-204 peripheral challenge mouse model. Antiviral Res (2012) 94(1):1–8 10.1016/j.antiviral.2012.02.001 22366350 PMC3331901

[44] Halstead SB Mahalingam S Marovich MA Ubol S Mosser DM Intrinsic antibody-dependent enhancement of microbial infection in macrophages: disease regulation by immune complexes. Lancet Infect Dis (2010) 10(10):712–22 10.1016/S1473-3099(10)70166-3 20883967 PMC3057165

[45] Thomas SJ Endy TP Critical issues in dengue vaccine development. Curr Opin Infect Dis (2011) 24(5):442–50 10.1097/QCO.0b013e32834a1b0b 21799408

[46] Bentsi-Enchill AD Schmitz J Edelman R Durbin A Roehrig JT Smith PG Long-term safety assessment of live attenuated tetravalent dengue vaccines: deliberations from a WHO technical consultation. Vaccine (2013) 31(23):2603–9 10.1016/j.vaccine.2013.03.038 23570986 PMC5355209

[47] Halstead SB Nimmannitya S Cohen SN Observations related to pathogenesis of dengue hemorrhagic fever. IV. Relation of disease severity to antibody response and virus recovered. Yale J Biol Med (1970) 42(5):311–28 5419206 PMC2591704

[48] Chen RT Markowitz LE Albrecht P Stewart JA Mofenson LM Preblud SR Measles antibody: reevaluation of protective titers. J Infect Dis (1990) 162(5):1036–42 10.1093/infdis/162.5.1036 2230231

[49] Amanna IJ Carlson NE Slifka MK Duration of humoral immunity to common viral and vaccine antigens. N Engl J Med (2007) 357(19):1903–15 10.1056/NEJMoa066092 17989383

[50] Misegades LK Winter K Harriman K Talarico J Messonnier NE Clark TA Association of childhood pertussis with receipt of 5 doses of pertussis vaccine by time since last vaccine dose, California, 2010. JAMA (2012) 308(20):2126–32 10.1001/jama.2012.14939 23188029

[51] Chaves SS Gargiullo P Zhang JX Civen R Guris D Mascola L Loss of vaccine-induced immunity to varicella over time. N Engl J Med (2007) 356(11):1121–9 10.1056/NEJMoa064040 17360990

[52] Morrison D Legg TJ Billings CW Forrat R Yoksan S Lang J A novel tetravalent dengue vaccine is well tolerated and immunogenic against all 4 serotypes in flavivirus-naive adults. J Infect Dis (2010) 201(3):370–7 10.1086/649916 20059357

[53] Slifka MK Amanna IJ How advances in immunology provide insight into improving vaccine efficacy. Vaccine (2014). 10.1016/j.vaccine.2014.03.078 24709587 PMC4096845

[54] Niedrig M Lademann M Emmerich P Lafrenz M Assessment of IgG antibodies against yellow fever virus after vaccination with 17D by different assays: neutralization test, haemagglutination inhibition test, immunofluorescence assay and ELISA. Trop Med Int Health (1999) 4(12):867–71 10.1046/j.1365-3156.1999.00496.x 10632996

[55] Poland JD Calisher CH Monath TP Downs WG Murphy K Persistence of neutralizing antibody 30-35 years after immunization with 17D yellow fever vaccine. Bull World Health Organ (1981) 59(6):895–900 6978196 PMC2396120

[56] Bonsignori M Moody MA Parks RJ Holl TM Kelsoe G Hicks CB HIV-1 envelope induces memory B cell responses that correlate with plasma antibody levels after envelope gp120 protein vaccination or HIV-1 infection. J Immunol (2009) 183(4):2708–17 10.4049/jimmunol.0901068 19625640 PMC3089979

[57] Mason RA Tauraso NM Spertzel RO Ginn RK Yellow fever vaccine: direct challenge of monkeys given graded doses of 17D vaccine. Appl Microbiol (1973) 25(4):538–44 4633476 10.1128/am.25.4.539-544.1973 PMC380857

[58] Sun W Eckels KH Putnak JR Lyons AG Thomas SJ Vaughn DW Experimental dengue virus challenge of human subjects previously vaccinated with live attenuated tetravalent dengue vaccines. J Infect Dis (2013) 207(5):700–8 10.1093/infdis/jis744 23225894

[59] Thomas SJ Dengue human infection model: re-establishing a tool for understanding dengue immunology and advancing vaccine development. Hum Vaccin Immunother (2013) 9(7):1587–90 10.4161/hv.24188 23466948

[60] Mammen MP Lyons A Innis BL Sun W McKinney D Chung RC Evaluation of dengue virus strains for human challenge studies. Vaccine (2014) 32(13):1488–94 10.1016/j.vaccine.2013.12.040 24468542

[61] Durbin AP Whitehead SS The dengue human challenge model: has the time come to accept this challenge? J Infect Dis (2013) 207(5):697–9 10.1093/infdis/jis749 23225898

[62] Amanna IJ Slifka MK Wanted, dead or alive: new viral vaccines. Antiviral Res (2009) 84(2):119–30 10.1016/j.antiviral.2009.08.008 19733596 PMC2760379

